# Laryngeal Stenosis

**Published:** 1896-02

**Authors:** W. Jay Bell

**Affiliations:** Atlanta, Ga.; Assistant to the Chair of Obstetrics and Gynecology Southern Medical College


### LARYNGEAL STENOSIS.

By W. JAY BELL, A.B., M.D., Atlanta, Ga.

Assistant to the Chair of Obstetrics and Gynecology Southern Medical College.

It is not my purpose in this article to discuss either diphtheria or membranous croup, yet these must be mentioned as the most frequent causes of laryngeal stenosis. In laryngeal diphtheria the stenosis is due to either membranous encroachment upon the laryngeal caliber or to inflammatory or œdematous swellings.

Diphtheria antitoxin, however efficacious, will not relieve this factor of danger in diphtheria. Antitoxin acts through physiological routes, and notwithstanding its degenerative power upon pseudo-membrane, its action is entirely too slow for laryngeal stenosis, and resting on the oars of antitoxin will waft the little patient into eternity. I would not be misunderstood in this, for diphtheria antitoxin has been so fully demonstrated to be efficacious both as a curative and as an immunizing agent, that a fatal issue following a failure to use this remedy is to be severely censured by the profession.

Yet stenosis is of a mechanical nature, and cannot be relieved by an antitoxic. Croup—spasmodic or membranous—or œdema, may give serious laryngeal obstruction. In diphtheria, membranous croup, or in œdema, relaxants and antispasmodics are absolutely futile, and are susceptible of great harm, as they depress and impair the vital forces, which should always be avoided. In spasmodic croup relaxants and antispasmodics may be used with great benefit.

Confronted with a case of laryngeal stenosis, not spasmodic in character, the question arises: What steps must be taken? And frequently immediate steps are demanded. Well, there is but one thing demanded; that is, mechanical relief of the stenosis. At this point a determination of the nature of the interference: Shall it be *tracheotomy* or *intubation?* Tracheotomy means *preparation, consent of parents, blood, shock;* all of which are out of the question where an immediate interference is demanded.

Then, too, what hope have you should your warning be sufficient to allow a tracheotomy? A fatal issue is the usual result. Not quite a contraindication, yet a great objection, is the horror of a mother to consent that her child's throat should be cut.

*Intubation* means a clean intubating set, no preparation, no blood, no shock, and only a few minutes' time, with instantaneous relief.

It may be held by some that the obstruction may extend below the larynx. Well, experience shows that it very rarely does, and if so tracheotomy can be done with greater ease and facility with the tube in place, as the tube will give at least partial relief, and will serve as a guide to the trachea below. A guide may seem unnecessary, and would be in adults, but in a child the rings are so soft that at times difficulty is experienced in identifying the trachea, and anything that will help to identify the trachea will serve a good purpose. Intubation is not a difficult operation, and is often done in seven to ten seconds after the gag is in place.

Dillon Brown is one of the most expert intubators in New York City, and during my stay in New York I had the honor of being with him at several intubations, and had the pleasure of taking a course under him during last year.

During my service in the New York Infant Asylum and Hospital there was an epidemic of diphtheria, and I had occasion several times to do intubation. Tracheotomy was done in several cases, with fatal issue in each, while there were only two cases fatal after intubation, one due to accident and one to heart-failure.

Intubation offers the most immediate, and most absolute, and most satisfactory relief, with still as favorable surroundings—yes, more favorable—for tracheotomy, the tube giving at least partial relief and serving as a guide to the trachea. I should always do intubation as first resort, and rarely will tracheotomy be necessary.

206 *Lee street, Atlanta.*

